# TMEM158 Regulates the Canonical and Non-Canonical Pathways of TGF-β to Mediate EMT in Triple-Negative Breast Cancer

**DOI:** 10.7150/jca.65822

**Published:** 2022-05-21

**Authors:** Jiaci Tong, Haoran Li, Ye Hu, ZuoWei Zhao, Man Li

**Affiliations:** Department of Oncology & Department of Breast Surgery, The Second Hospital of Dalian Medical University, Dalian, 116023 China

**Keywords:** Carcinogenesis, Triple-negative breast cancer, EMT, TGF-β signal pathway, TMEM158

## Abstract

Triple-negative breast cancer (TNBC) is a highly aggressive form of breast cancer with high metastatic potential. To date, no directed treatment options have been developed for the treatment of metastatic or advanced TNBC. The oncogene TMEM158, also known as RIS1, is upregulated by Ras-induced cellular senescence. Although TMEM158 has been shown to be involved in tumor progression, little is known about the molecular function and expression of TMEM158 in breast cancer. The present study evaluated the expression and prognostic relevance of TMEM158 in breast cancer patients from several databases. Gene set enrichment analysis (GSEA) showed that TMEM158 was closely associated with epithelial-mesenchymal transition (EMT) and TGF-β pathways. Gain- and loss-of-function assays indicated that overexpressed TMEM158 might participate in EMT by activating the TGF-β pathway, which in turn promotes tumor migration, invasion, and metastasis. These findings suggest that TMEM158 has the potential to become a new therapeutic target for TNBC.

## Introduction

Breast cancer is the second most frequent cause of cancer mortality in women worldwide 1. Triple-negative breast cancer (TNBC) is characterized by the absence of expression of estrogen receptor (ER) and progesterone receptor (PR), as well as the lack of amplification/overexpression of HER2 2. TNBC tends to be more aggressive, with earlier relapse potential and shorter relapse-free survival times than other breast cancer subtypes 2, 3. Because hormonal- and HER2-targeted therapies are ineffective in patients with TNBC, chemotherapy alone is the only approved treatment strategy for TNBC 4. Most patients with TNBC have a high risk of relapse and poor prognosis during the first 3-5 years after initial treatment 5. The identification of biomarkers and the elucidation of molecular mechanisms are therefore essential for the development of novel targeted agents and individualized therapy in patients with TNBC.

TMEM158, also known as the tumor suppressor RIS1, is upregulated during Ras-induced senescence in Ras-infected fibroblasts 6. TMEM158 was shown to be abnormally upregulated in pancreatic cancer and to be associated with poor clinical outcomes through its activation of the PI3K/AKT pathway 7. High expression of TMEM158 in ovarian cancer was found to be closely related to cell proliferation and invasion 8. The gene encoding TMEM158 is located on chromosome 3q21.3, the region with the highest frequency of heterozygosity loss in breast cancers 9, 10. Analysis of the TMEM158 gene in 60 patients with primary breast cancer showed no mutations 11. Furthermore, RT-PCR showed that TMEM158 was highly expressed in several TNBC cell lines, but that study did not evaluate protein expression or analyze the molecular features associated with increased TMEM158 expression 12. To investigate the role of TMEM158 in breast cancer, we determined its expression profile in various breast cancer cell subtypes. The effect of TMEM158 on the proliferation, adhesion and invasion of triple negative breast cancer cells was assessed. Possible mechanisms involved were also explored. Our study provides further insight into the role of TMEM158 in triple-negative breast cancer, which may be an effective therapeutic target for triple-negative breast cancer.

## Methods

### Bioinformatics analysis

The Cancer Genome Atlas (TCGA) (https://tcga-data.nci.nih.gov/tcga) was reviewed to obtain mRNA-seq data, phenotype, and survival profiles. Specifically, data on gene expression data of 1102 breast cancer tissue samples and 113 normal control breast tissue samples were obtained from the TCGA-breast cancer (BRCA) dataset. Differentially expressed genes (DEGs) in these two sets of tissues were identified and normalized using the R package "edgeR," with thresholds of P<0.05 and log FC >1.5.

To explore further the pathways associated with breast cancer pathogenesis, gene set enrichment analysis (GSEA) was performed using the TCGA-BRCA dataset.

### Breast cancer cell lines

The human breast cancer cell lines MCF-7, T47D, SKBR-3, HCC1954, HCC38, HCC1187, MDA-MB-231, BT549, MDA-MB-453, and HCC1937, and the non-tumorigenic human mammary gland cell line MCF-10A were obtained from the American Type Culture Collection (ATCC; Manassas, VA, USA). Ten cell lines of our included were subjected to westernblot assays to validate the expression profile of TMEM158 in various types of breast cancer cell lines. MDA-MB-231 cells were cultured in DMEM, and MCF-7, T47D, SKBR-3, HCC1954, HCC38, HCC1187, BT549, MDA-MB-453, and HCC1937 cells were cultured in RPMI1640, each containing fetal bovine serum (FBS, 10%), streptomycin (100 μg/ml), and penicillin (100 U/ml). MCF-10A cells were cultured in DMEM-F12 containing FBS (10%), penicillin (100 U/ml), streptomycin (100 μg/ml), insulin (10 µg/ml), hydrocortisone (0.5 µg/ml) and epidermal growth factor (EGF, 20 ng/ml). All cells were cultured at 37°C in a humidified incubator with 5% CO_2_, with medium changed every 1-3 days, based on the number of cells and their growth rates. Where indicated, MDA-MB-231 cells were pretreated overnight in the presence or absence of 20 μM PD98059 (MedChem Express, USA), an inhibitor of ERK1/2 signaling. Cells were harvested and assayed by western blotting.

### siRNA or overexpression plasmid transfection

The TMEM158 siRNAs 5'-GCCCUAGAUUCAUGGCAGATT-3' (siRNA1) and 5'-GGGAAGGAUUUAACACCGATT-3' (siRNA2) and the negative control siRNA 5'-UUCUCCGAACGUGUCACGUTT-3' (NC) were acquired from GenePharma. All siRNAs were transfected into cells using transfect-mate (GenePharma) according to the manufacturer's instructions.

BT549 and HCC1187 cells were seeded in six-well plates and incubated for 24 h until growing to 50% confluence. Each siRNA and the transfect-mate transfection reagents were diluted separately with serum-free medium, mixed, incubated for 20 min, and added dropwise to each well containing cells. The medium was exchanged after 24 h incubation. The effects of TMEM158 knockdown were analyzed by western blotting.

We introduced the TMEM158 overexpression plasmid into MDA-MB-231 to induce the expression of TMEM158. MDA-MB-231 cells were seeded in six-well plates and incubated for 24 h until growing to 80% confluence. Cells were transfected with 5 μg of overexpression plasmid or empty vector control, according to the manufacturer's instructions. Transfection efficiency was analyzed 72 h later by western blotting.

### Wound healing assays

BT549, HCC1187, and MDA-MB-231 cells were transfected for 24 h and plated in wells of six-well plates until the cells were 90% confluent. Cells in each well were scratched using 200 μl pipette tip, followed by the replacement with fresh medium without FBS and further incubation. Wells were photographed 0 h, 24 h, and 48 h after scratching, and the wound healing rate was calculated by measuring the scratch areas using Image J software. Percent wound healing was calculated as (S0-St)/S0.

### Migration and invasion assays

After transfection for 24 h, transwell assays were performed in 8 μm transwell chambers (Corning). Single-cell suspensions of BT549 (12 × 10^4^ cells/200 μl), HCC1187 (7 × 10^3^ cells/200 μl), and MDA-MB-231 (5 × 10^4^ cells/200 μl) cells in serum-free medium were added to the upper chambers of transwell plates, and 800 μl of medium containing 15% FBS were added to each lower chamber and the plates incubated for 48 h, followed by staining with 0.5% crystal violet for 20 min. The chambers were washed with PBS, wiped clean with a cotton swab, and allowed to dry before being visualized, photographed, and counted with an inverted microscope.

To perform invasion assays, serum-free medium and matrigel were mixed at a ratio of 8:1, and 40 μl were applied evenly to the upper chambers of transwell plates, each of which contained 5 × 10^4^ cells in 200 μl medium, followed by incubation overnight. Aliquots containing 800 μl of medium containing 15% FBS were added to the lower chambers and the plates incubated for 48 h. Cells were stained with 0.5% crystal violet for 20 min, visualized, photographed, and counted as described above.

### Cell viability assays

BT549, HCC1187, and MDA-MB-231 cells were transfected for 24 h, followed by the transfer of 5 × 10^3^, of 2 × 10^3^, and 8 × 10^3^ cells, respectively, into each well of a 96-well plate. CCK-8 was added at specific time points to each well, followed by incubation for 1-4 h, and measurement of OD at 490 nm with a microplate reader (Bio-Rad). Each assay was repeated at least three times.

### Colony formation

Aliquots of 5 × 10^3^ cells were seeded into each well of a 6-well plate, followed by incubation in a humidified atmosphere at 37°C with 5% CO_2_ for 7-14 days until colonies of cells appeared. Colonies were stained with 0.5% crystal violet and counted.

### Western blotting

Cells were lysed in a buffer containing phenylmethylsulfonyl fluoride and phosphatase and protease inhibitors. The lysates were centrifuged, and the protein concentrations in the supernatants assessed by the BCA method. Total proteins were electrophoresed on SDS-polyacrylamide gels and transferred onto nitrocellulose membranes (GE Healthcare Life Science). The membranes were blocked by incubation in 5% nonfat milk for 1 h, followed by incubation with primary antibodies overnight at 4°C. The primary antibodies utilized in this study included anti-TMEM158 (Abcam, Cat# ab98335), anti-ERK (CST, Cat# ab4695S), anti-p-ERK (CST, Cat# ab4370S), anti-vinculin (Abcam, Cat# ab129002), anti-E-cadherin (Proteintech, Cat# 10366-1-AP), anti-N-cadherin (Proteintech, Cat# 22018-1-AP), anti-vimentin (Proteintech, Cat# 10366-1-AP), anti-TGFβ1 (Wanleibio, Cat# WL02998), anti-ZEB1 (Wanleibio, Cat# WL03489), anti-SNAIL (Wanleibio, Cat# WL01863), Anti-Twist1 (Wanleibio, Cat# WL03489), anti-t-SMAD2/3 (Wanleibio, Cat# WL01520), and anti-p-SMAD2/3 (Wanleibio, Cat# WL02305) antibodies. The levels of expression of these proteins were normalized relative to the expression of vinculin in the same samples.

### Statistical analysis

Data obtained from the TCGA database were analyzed using R software (version 4.0.1, http://www.r-project.org/). Results were presented as mean ± SEM. Differences between two groups were assessed using paired/independent Student's t tests and differences among three or more groups by analysis of variance (ANOVA). Survival was assessed by the Kaplan-Meier method and compared by log-rank tests. Histograms were plotted using Graphpad Prism 8.0 software. P values <0.05 were considered statistically significant.

## Results

### The expression of TMEM158 is aberrant in TNBC tissues from databases

**Figure 1:**
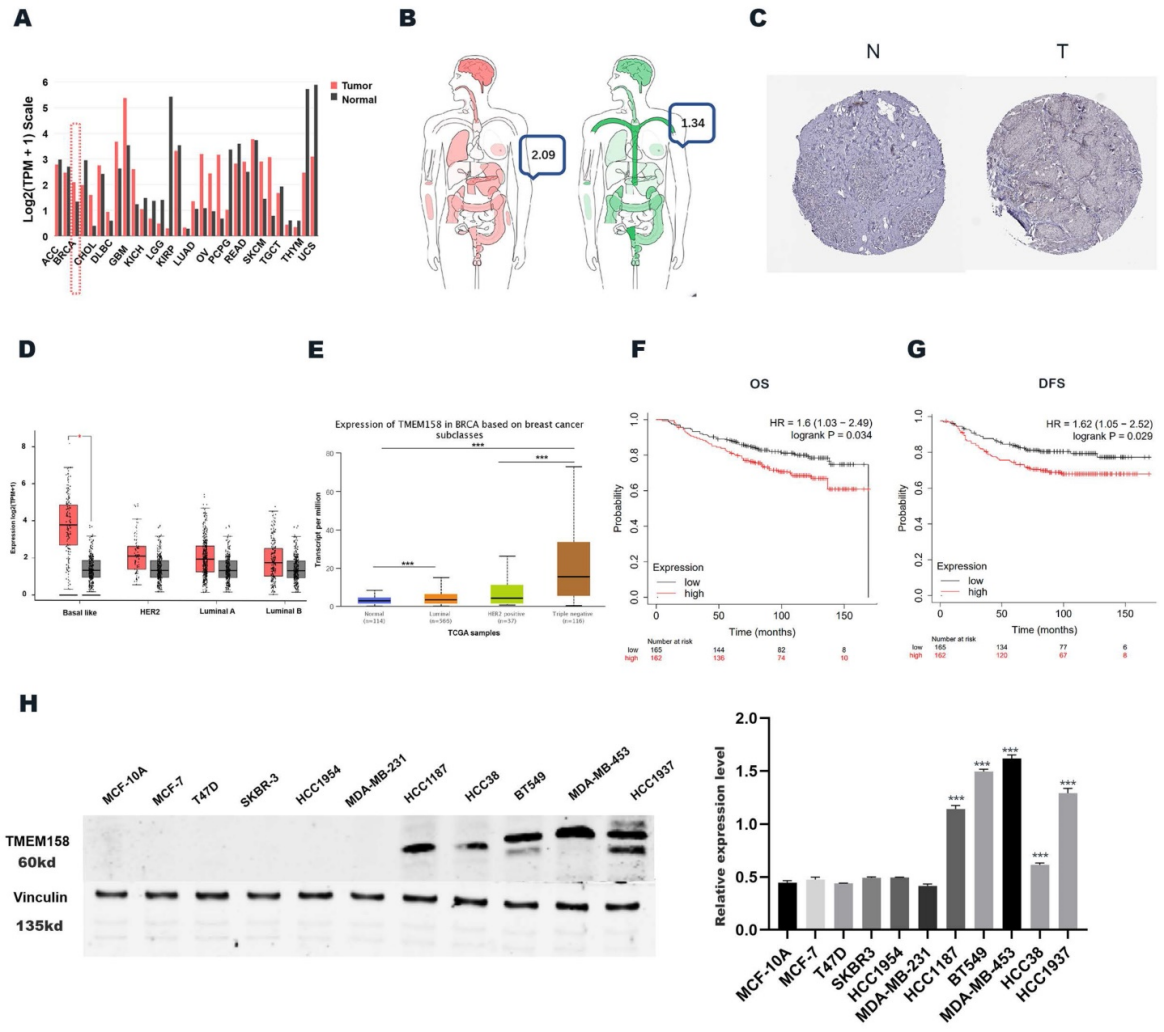
Triple-negative breast cancers express high levels of TMEME158. (A) and (B) TMEM158 expression is upregulated in breast cancer compared to normal tissues in GEPIA Database. (C) Comparison between protein expression of TMEM158 in BC and normal tissues in Human Protein Atlas (HPA). TMEM158 protein expression in breast cancer tissues was elevated compared to that in normal tissues. (D) and (E) Expression of TMEM158 in each breast cancer subtype. TMEM158 has a high expression level in triple-negative breast cancer compared to non-triple-negative breast cancer. (F) and (G) TMEM158 was highly expressed in TNBC and related to a poor clinical outcome from GSE20685 gene set. (H) Protein expression profiles of TMEM158 in various breast cancer cell lines. TMEM158 expression was analyzed by western blotting in MCF-7, T47D, SKBR-3, HCC1954, HCC38, HCC1187, MDA-MB-231, BT549, MDA-MB-453 and HCC1937, and the human mammary gland cell line MCF-10A. The bars indicate quantification of protein. N: normal tissue; T: tumor tissue. *p<0.05, **p<0.01, ***p<0.001, ns: nonsignificant

The levels of expression of TMEM158 mRNA were compared in breast cancer and normal tissues in the Gene Expression Profiling Interactive Analysis (GEPIA) (http://gepia.cancer-pku.cn) and UALCAN (http://ualcan.path.uab.edu/) databases (Figures 1A, 1B). We obtained immunohistochemistry results from the HPA database (https://www.proteinatlas.org) (Figure 1C) to validate our conjecture that BC expressed higher levels of TMEM158 than that of normal tissues. Analysis showed that TMEM158 mRNA levels were higher in tumor tissues from patients with TNBC than in tumor tissues from patients with other types of breast cancer and from normal breast tissue (Figures 1D, 1E).

### Prognostic significance of TMEM158 for TNBC patients

The GSE20685 dataset was analyzed using the publicly accessible Kaplan-Meier plotter online database (http://kmplot.com/) to confirm the clinical importance of TMEM158 in patients with TNBC and to determine the correlations between TMEM158 expression levels and patient survival and prognosis. Lower expression of TMEM158 was associated with better overall survival (OS) and disease-free survival (DFS) in patients with TNBC (Figures 1F, 1G).

### TMEM158 knockdown represses invasiveness, migration, and proliferation of TNBC cells

**Figure 2:**
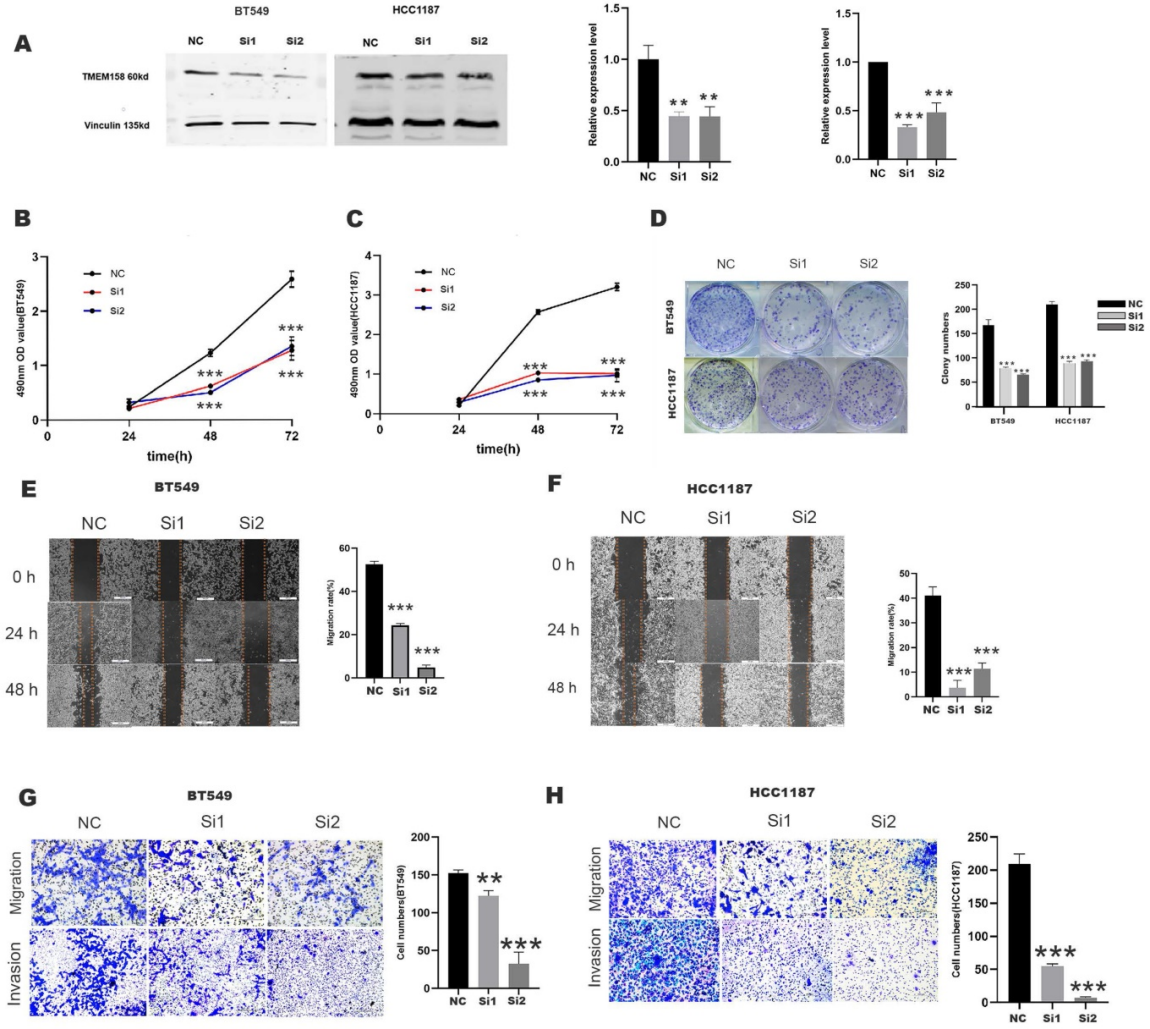
TMEM158 downregulation reduces the aggressivity of TNBC cells in vitro. (A) Western blotting showing that transfection of specific siRNAs knocked down TMEM158 expression in BT549 and HCC1187 cells; the bar graph shows protein quantification. (B) and (C) TMEM158 is involved in tumor proliferation. Cell Counting Kit-8 assays measuring the proliferative capacity of siRNA-transfected BT549 and HCC1187 cells 24 h, 48 h, and 72 h after seeding. (D) Knockdown of TMEM158 attenuates the proliferation of breast cancer cells in vitro. Colony formation assays showing the relative proliferation capacity (left) and numbers of colonies numbers (right) of BT549 and HCC1187 cells transfected with specific TMEM158 siRNAs. (E) and (F) Tumor migration is associated with aberrant expression of TMEM158. BT549 and HCC1187 cells transfected with specific TMEM158 siRNAs and grown to 90% confluence were scratched with a 200 μl pipette tip, and incubated in serum-free medium for 48 h. Cells were photographed at 0 h, 24 h and 48 h to assess the rates of wound closure. The bar graphs show the means ± SEM of three independent experiments. (G) and (H) Transwell migration and invasion assays, showing that TMEM158 facilitates cell migration and invasion (left) and cell quantification (right). NC: negative control siRNA; Si1: TMEM158 siRNA1; Si2: TMEM158 siRNA2; CCK‐8: Cell Counting Kit‐8; SD: standard deviation. All data are shown as mean ± SD. All experiments were repeated at least three times. *p<0.05, **p<0.01, ***p<0.001, ns: nonsignificant. Upregulation of TMEM158 promotes PC cell aggressiveness in vitro.

Evaluation of TMEM158 expression in 10 breast cancer cell lines showed that TMEM158 was overexpressed in BT549 and HCC1187 cells, but was not expressed in MDA-MB-231 cells (Figures 1H). To determine the biological functions of TMEM158 in TNBC cell lines, TMEM158 was knocked down in BT549 and HCC1187 by transfection of siRNA, the expression efficiency was verified by Western blot assay (Figures 2A). Both TMEM158 siRNAs, siRNA1 and siRNA2, significantly inhibited the expression of TMEM158, and transfection of the TMEM158 overexpressing plasmid into MDA-MB-231 cells resulted in the expression of the protein. Both siRNA1 and siRNA2 significantly reduced the proliferation of BT549 and HCC1187 cells (Figures 2B, 2C) and significantly reduced colony formation by BT549 and HCC1187 cells (Figure 2D). Wound healing assays showed that transfection of both siRNA1 and siRNA2 into BT549 and HCC1187 cells significantly reduced wound closure rates (Figures 2E, 2F). Matrigel transwell assays evaluating the potential roles of TMEM158 in tumor invasion and migration showed that both siRNA1 and siRNA2 significantly reduced BT549 and HCC1187 cell migration and invasion (Figures 2G, 2H).

### Upregulation of TMEM158 promotes invasive, migratory and proliferative properties in MDA-MB-231 cells

**Figure 3:**
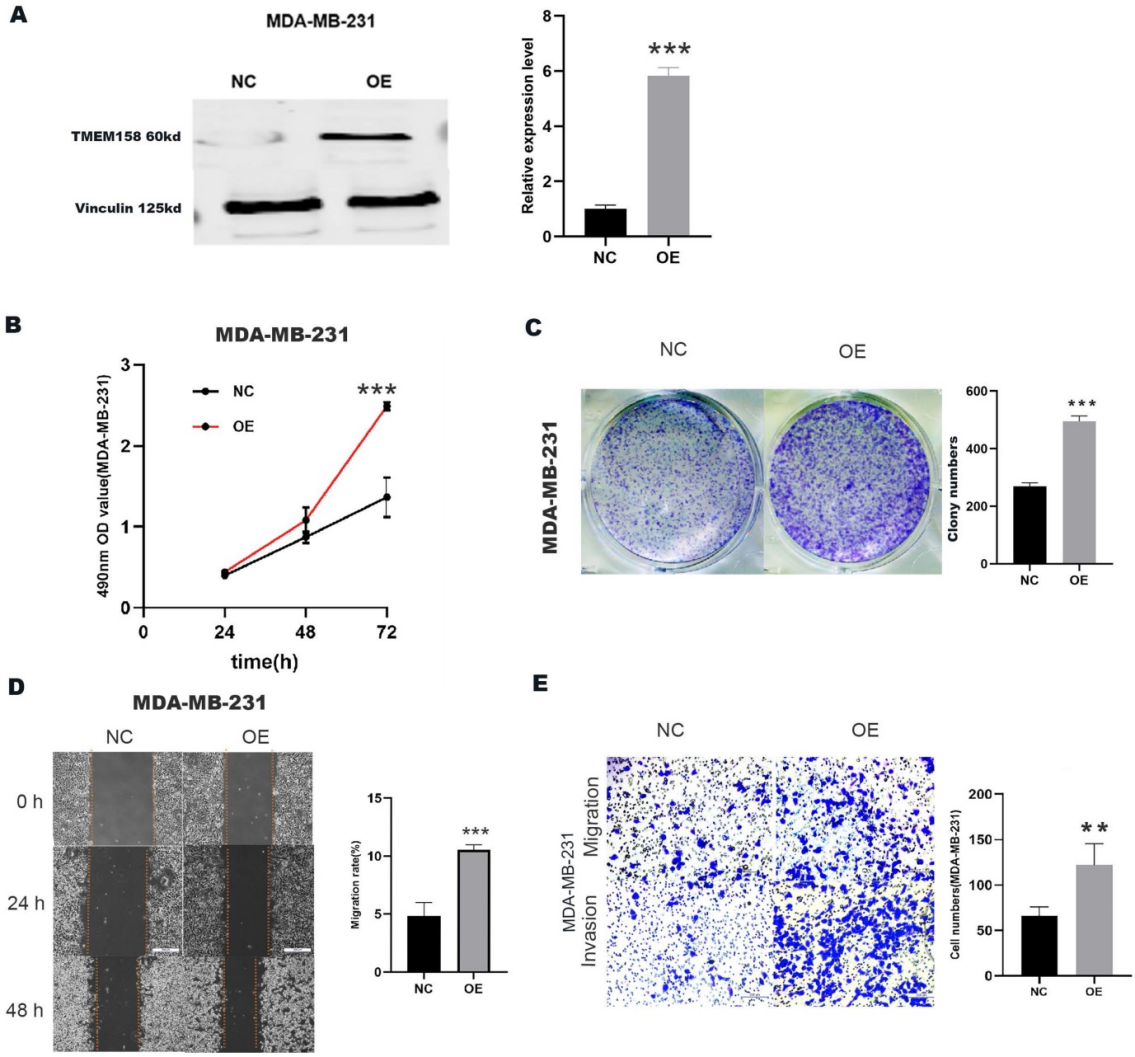
Upregulation of TMEM158 promotes TNBC cells aggressiveness in vitro. (A) Western blot confirmation showing that transfection of a plasmid overexpressing TMEM158 resulted in its overexpression in MDA-MB-231 cells. (B) Cell Counting Kit-8 assays of the viability of MDA-MB-231 cells after transfection with OE or NC plasmid as indicated. (C) Colony formation assays of MDA-MB-231 cells after transfection with OE or NC plasmid as indicated. (D) Wound healing assay comparing the migration potential of MDA-MB-231 cells after transfection with OE or NC plasmid as indicated. (E) Transwell migration and invasion assays of MDA-MB-231 cells after transfection with OE or NC plasmid as indicated. NC: negative control; OE: overexpression; CCK‐8: Cell Counting Kit‐8; SD: standard deviation. All data are shown as mean ± SD. All experiments were repeated at least three times. *p<0.05, **p<0.01, ***p<0.001, ns: nonsignificant.

A plasmid overexpressing TMEM158 was transfected into MDA-MB-231 cells, the expression efficiency was verified by Western blot assay (Figure 3A). Compared to the control group, transfection of the TMEM158 overexpressing plasmid significantly enhanced cell proliferation (Figure 3B) and colony formation (Figure 3C) in MDA-MB-231 cells. Furthermore, upregulation of TMEM158 significantly stimulated migration and invasion of MDA-MB-231 cells, as confirmed by wound healing assays demonstrated this (Figure 3D) and matrigel transwell (Figure 3E).

Taken together, all findings support an important role for TMEM158 in promoting cell proliferation, migration and invasion.

### TMEM158 facilitates the EMT process

**Figure 4:**
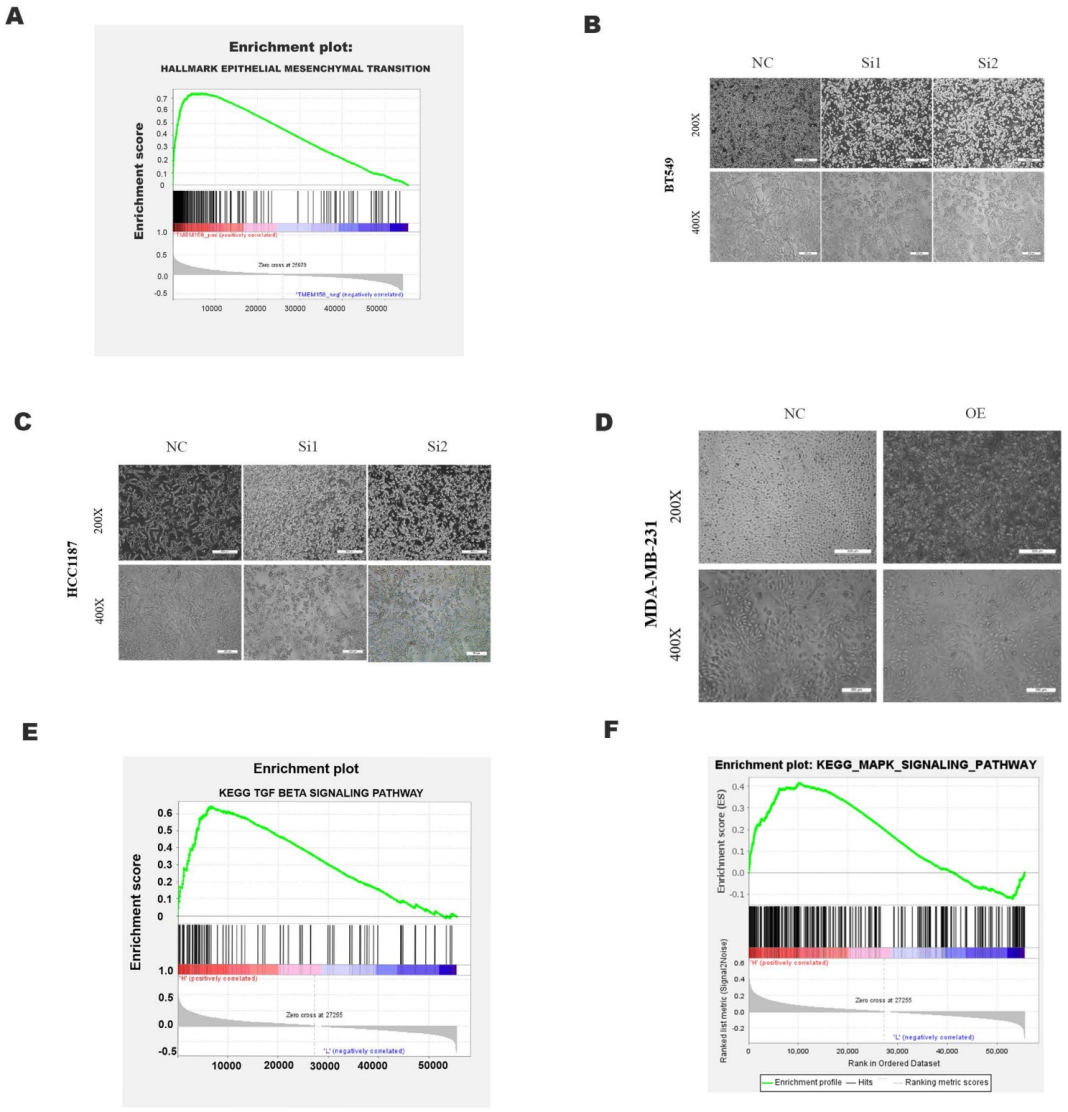
Effects of TMEM158 on tumorigenesis and progression. GSEA analysis showing the association between TMEM158 and EMT. (B)-(D) Effects of TMEM158 knockdown in BT549 and HCC1187 cells and overexpression in MDA-MB-231 cells on cell morphology. (E)-(F) GSEA enrichment analysis, showing the association of TMEM158 with the TGF-β signaling pathway and MAPK signaling pathway. NC: negative control siRNA; Si1: TMEM158 siRNA1; Si2: TMEM158 siRNA2; OE: TMEM158 overexpression; SD: standard deviation. All data are shown as mean ± SD. All experiments were repeated at least three times. *p<0.05, **p<0.01, ***p<0.001, ns: nonsignificant

**Figure 5:**
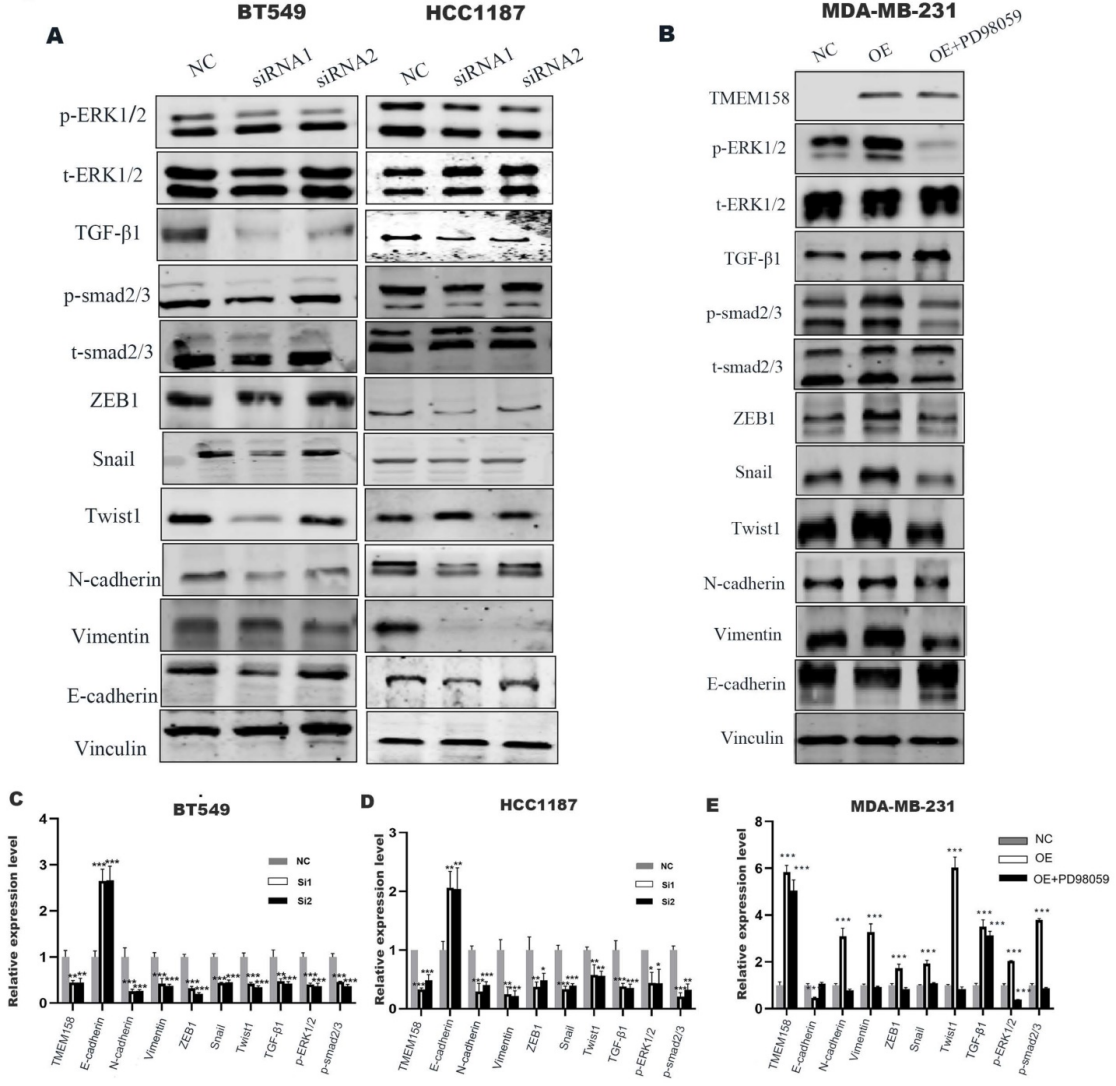
TMEM158 triggers EMT by activating the canonical and non-canonical TGF-β signaling pathways. (A) Western blot analysis of the TGF-β signaling pathway, phosphorylated ERK1/2, total ERK1/2 and epithelial/mesenchymal protein marker, in BT549 and HCC1187 cells. (B) Effect of the ERK phosphorylation inhibitor PD98059 on ERK signaling in TMEM158 overexpressing MDA-MB-231 cells. Western blotting analysis of proteins in the Smad-dependent pathway, phosphorylated ERK1/2, total ERK1/2 and epithelial/mesenchymal protein markers; the bar graph shows protein quantification. (C)-(E) The bar graphs show protein quantification. NC: negative control siRNA; Si1: TMEM158 siRNA1; Si2: TMEM158 siRNA2; OE: TMEM158 overexpression. *p<0.05, **p<0.01, ***p<0.001, ns: nonsignificant

GSEA analysis using the TCGA dataset showed a significant positive correlation between TMEM158 expression and EMT (Figure 4A). The EMT process involves alterations in cell morphology, increases in cell motility and reductions in cell-to-cell contacts, enhancing the tumor cell migration and invasion. EMT also results in the lack of expression and function of epithelial markers, such as E-cadherin, and the upregulation of mesenchymal cell markers, such as N-cadherin and vimentin 13. Knockdown of TMEM158 in HCC1187 and BT549 cells altered their morphology, with cells becoming shorter and rounder, assuming epithelial-like features. TMEM158 overexpression in MDA-MB-231 cells also altered their morphology, with the cells becoming longer and showing a large number of fibroblast-like features (Figures 4B-D). Western blotting showed that knockdown of TMEM158 downregulated the expression of N-cadherin and vimentin while upregulating the expression of E-cadherin, whereas TMEM158 overexpression had the opposite effects (Figure 5A and B). The regulatory network formed by EMT transcription factors includes a variety of interacting proteins, which contribute to a strong transcriptional control of EMT 14. Additional assays revealed that the EMT transcription factors, such as ZEB1, SNAIL, and Twist1, associated with TMEM158 were indicative of mesenchymal cell markers (Figure 5A and B).

### TMEM158 triggers EMT through activated canonical and non-canonical TGF-β signaling pathways

GSEA analyses of this dataset were performed to identify the gene set associated with TNBC. Based on its normalized enrichment score, the TGF-β and MAPK pathways were found to be closely related to this gene set (Figure 4E-F). The TGF-β signaling pathways were previously shown to play a significant role in EMT 15, with these pathways being both Smad-dependent and Smad-independent 16. For the latter, TGF-β can launch the MAPK/ERK pathway, PI3K/Akt pathway and Wnt/β-catenin pathway to upregulate the levels of EMT-TFs 17. In addition, ERK has been shown to rapidly activate the TGF-β pathway independent of Smad 18. Knockdown of TMEM158 in HCC1187 and BT549 cells was found to markedly reduced the expression of TGF-β1, p-smad2/3, and p-ERK (Figure 5A).

To confirm the role of the ERK1/2 signaling pathway in TMEM158-mediated EMT progression, ERK signaling was inhibited in MDA-MB-231 cells overexpressing TMEM158 by downregulating phosphorylated ERK1/2 using the ERK phosphorylation inhibitor PD98059. Western blotting showed that TMEM158 overexpression inhibited ERK1/2 signaling in MDA-MB-231 cells, with downregulation of the ERK1/2 signaling pathway inhibiting the TGF pathway (Figure 5B). Although PD98059 did not alter the level of TGF-β1 expression, it reduced the expression of p-smad2/3 and EMT-related transcription factors, while enhancing the expression of E-cadherin. Taken together, these findings indicate TMEM158 is involved in tumor invasion and metastasis regulated by EMT through activation of both the canonical and non-canonical TGF-β signaling pathways.

## Discussion

TNBC is characterized by poor prognosis and high recurrence, metastasis, and fatality rates 3, 19. The present study revealed that TMEM158 was aberrantly expressed in TNBCs and correlated with poor clinical outcomes and prognosis. TMEM158 belongs to the TMEM family 20, 21. TMEMs are present in many types of cells and are involved in various biological functions, including smooth muscle contraction 22, autophagy, and epidermal keratinization 23. However, TMEMs were also found to modulate tumor cell dissemination and drive tumor metastasis 21. TMEM158 gene expression is closely associated with the progression of ovarian and pancreatic cancers, but was not known to be involved in breast cancer. To our knowledge, this study is the first to show that TMEM158 is upregulated in breast cancer and contributes to tumorigenesis and progression.

TMEM158 was previously regarded as a tumor suppressor, due in part to its involvement in Ras-induced cellular senescence in fibroblasts 6, which is regarded as an anti-tumor response 24, 25. In addition, TMEM158 is located on chromosome 3p21.3, a chromosomal region frequent lost in many types of cancer, suggesting that this region is likely contains multiple tumor suppressor genes 26-28. Quantification of TMEM158 expression in tumor and normal tissue samples of 60 individuals with primary breast cancer showed that, although TMEM58 expression is suppressed in 23% of tumors, it was upregulated in 15% 11, suggesting it may not be an oncogene in breast cancer. TMEM158 was shown to be overexpressed in pancreatic, ovarian, and non-small cell lung cancers 29, and high expression of TMEM158 in colorectal cancer cells was found to promote drug resistance 30. These findings suggest that TMEM158 may have multiple functions depending on the type of cancer. The present study confirmed its expression profile in TNBC, showing that TMEM158 overexpression mediates EMT by mobilizing the TGF-β pathway and thereby participating in tumor adhesion and invasion.

EMT is a multistep reversible process that modifies cell morphology and behavior 14. Epithelial cells undergoing EMT experience a series of structural changes, such as loss of polarity, reduced contact with surrounding tissues and cells, and enhanced migration. These cells also experience an alteration in phenotype, with a gradual loss of epithelial properties (e.g., E-cadherin expression) and an increase in mesenchymal properties (e.g., vimentin and N-cadherin expression) 31, 32. The intensity of EMT is mainly dependent on the potency of transcription factors (e.g., ZEB1, Twist1, SNAIL) that trigger cellular reprogramming 14, 33-35. In general, transcriptionally regulated proteins form distinctive regulatory networks during EMT. Aberrant activation of EMT by cancer cells, which leads to enhanced aggressiveness and tumor tissue motility, is considered a key event in tumor progression 36. GSEA analysis showed that the gene set that includes TMEM158 was associated with EMT. Knock down of TMEM158 was found to reduce the expression of N-cadherin and vimentin, while enhancing the expression of E-cadherin. These findings suggest that, by promoting EMT, TMEM158 may be engaged in tumor invasion and metastasis.

The intensity of EMT is modulated by intra- and extracellular signaling mechanisms, with TGF-β being a master regulator of EMT 37. Treatment of relatively non-invasive breast cancer cells with TGF-β enhanced their invasiveness, as well as increasing the intensity of EMT 37, suggesting that TGF-β drives the EMT of cancer cells to increase their metastatic ability 38. GSEA analysis of the TCGA-BRCA dataset showed that the TGF-β and MAPK signal pathway gene set was tightly linked to TNBC, and that the genes in this set were closely associated with TMEM158. Moreover, survival analysis showed that high expression of TMEM158 correlated with poor clinical outcome and prognosis. These results suggest that TMEM158 is likely involved in the development and progression of TNBC via the TGF-β pathway.

The TGF-β signaling pathway has been shown to regulate cell proliferation, differentiation, migration and apoptosis through both Smad-dependent and Smad-independent pathways 39-41. Both pathways interact and integrate with each other to form a network that promotes tumorigenesis and progression. TGF-β interacts with two types of transmembrane receptors, TβRI and TβRII, to induce Smad-dependent signaling. Binding of TGFβ with TβRI and TβRII on cell membranes results in the formation of ligand-receptor complexes, followed by clustering and activation of Smad2/3. Phosphorylated Smad2/3 binds to Smad4 to form a complex, which undergoes nuclear translocation 42-44. Whereas the Smad pathway is crucial for TGF-β signalling, activated TGF-β receptors can also initiate potentially Smad independent pathways. Activated TGF-β can initiate the ERK/MAPK pathway by recruiting the junction Shc to a tyrosine phosphorylation residue with TβRI. The junction Grb2 complexed to Sos1 (the nucleotide exchange protein of Ras) can subsequently bind to tyrosine phosphorylation residues on Shc and activated Ras mediates activation of the ERK/MAPK pathway 45. The present study found that blocking the ERK signaling pathway could significantly reverse the EMT process induced by TMEM158 overexpression through the Smad-dependent pathway. Taken together, these findings indicate that upregulation of TMEM158 promotes TNBC proliferation, migration and invasion through activation of both the canonical and non-canonical pathways.

In conclusion, the present study is the first to show that TMEM158 is a critical gene in TNBC and a potential biomarker for its diagnosis, treatment, and prognostic surveillance. Bioinformatics analysis revealed that TMEM158 was overexpressed in TNBC and related to tumor progression. Further in vitro assays revealed that TMEM158 promotes TNBC development and progression by facilitating TGF-β-induced EMT. These results may enable better risk stratification, diagnosis and individualized treatment of patients with TNBC.

## Abbreviations

TNBC: triple-negative breast cancer
TMEM158: transmembrane protein 158
RIS1: ras-induced senescence 1
EMT: epithelial-mesenchymal transition
BC: breast cancer
ER: estrogen receptors
LOH: loss of heterozygosity
TCGA: The Cancer Genome Atlas
HPA: Human Protein Atlas
GSEA: gene set enrichment analysis
ATCC: American Type Culture Collection
DMEM: Dulbecco's modified Eagle's medium
DMEM-F12: Dulbecco's modified Eagle's medium F12
EGF: epidermal growth factor
NC: negative control
CCK-8: Cell Counting Kit-8
OD: optical density
OS: overall survival
DFS: disease-free progression survival
NES: normalized enrichment score
TMEM: transmembrane protein
TβRI: TGFβ type I receptors
TβRII: TGFβ type II receptors
